# Detection of Zika Virus in Urine

**DOI:** 10.3201/eid2101.140894

**Published:** 2015-01

**Authors:** Ann-Claire Gourinat, Olivia O’Connor, Elodie Calvez, Cyrille Goarant, Myrielle Dupont-Rouzeyrol

**Affiliations:** Institut Pasteur, Noumea, New Caledonia

**Keywords:** Zika virus, viruses, arboviruses, emerging disease, serum, urine, diagnosis, French Polynesia

## Abstract

We describe the kinetics of Zika virus (ZIKV) detection in serum and urine samples of 6 patients. Urine samples were positive for ZIKV >10 days after onset of disease, which was a notably longer period than for serum samples. This finding supports the conclusion that urine samples are useful for diagnosis of ZIKV infections.

Zika virus (ZIKV) is an emerging mosquito-borne pathogen (family *Flaviviridae*, genus *Flavivirus*) that was isolated in 1947 from a rhesus monkey in the Zika forest in Uganda (*1*). ZIKV is believed to be transmitted to humans by infected *Aedes* spp. mosquitoes (*2**,**3*). Studies have demonstrated that ZIKV is endemic to Africa and Southeast Asia (*4*). Before 2007, few cases of human infection with ZIKV had been reported. In 2007, an epidemic of ZIKV infection in humans occurred in Yap, Federated States of Micronesia, in the Pacific region. A seroprevalence survey determined that ≤70% of the population had been infected (*5*). During 2007–2013, the few cases of infection with ZIKV reported were in travelers returning from Africa (*6*) or Southeast Asia (*7*).

In humans, ZIKV infection is characterized by mild fever (37.8°C–38.5°C); arthralgia, notably of small joints of hands and feet; myalgia, headache; retroorbital pain; conjunctivitis; and cutaneous maculopapular rash. ZIKV infection is believed to be asymptomatic or mildly symptomatic in most cases (*5*). Thus, Zika can be misdiagnosed during the acute (viremic) phase because of nonspecific influenza-like signs and symptoms. Hemorrhagic signs have not been reported in ZIKV-infected patients (*5**–**7*). However neurologic complications, including Guillain-Barré syndrome, have been observed (*8*).

Biological confirmation of ZIKV infections is based mostly on detection of virus RNA in serum by using reverse transcription PCR (RT-PCR). Although IgM against ZIKV can be detected by ELISA, few laboratories have this ability. Thus, in addition to the nonspecific clinical features of infection with ZIKV, laboratory diagnosis is challenging because of low viremia and cross-reactivity of ZIKV antibodies with other flaviviruses (including dengue), which require confirmation by neutralization assays (*8*) and make rapid serologic confirmation difficult. We investigated the diagnostic utility of urine as a source for detection of ZIKV RNA by real-time RT-PCR.

## The Study

In October 2013, a ZIKV outbreak was reported in French Polynesia (*9*). This was the second outbreak of ZIKV infection reported in the Pacific region. In New Caledonia, where ZIKV infection had never been documented, the first cases of ZIKV infection imported from French Polynesia were confirmed by the end of November, and the first autochthonous cases were reported by mid-January 2014. Early in February 2014, the New Caledonia Health Authority declared an outbreak situation. By the end of August 2014, >1,400 cases of ZIKV infection were biologically confirmed, including 34 cases imported from French Polynesia (*10*).

Written informed consent was obtained from all patients in this study. Clinical signs and symptoms of 6 ZIKV-infected patients are shown in the Table. In this study, a cutaneous maculopapular rash of the trunk and extremities was systematically observed and considered a relevant clinical criterion. Complete blood counts showed a discreet perturbation common in many viral infections (mild leukopenia and thrombocytopenia associated with activated lymphocytes).

**Table:** Characteristics of 6 patients infected with Zika virus, New Caledonia, 2014*

Characteristics	Patient 1	Patient 2	Patient 3	Patient 4	Patient 5	Patient 6
Clinical						
Headache	–	–	–	–	+	–
Arthralgia	–	+++ (ankles)	++ (hands)	+++	++ (hands)	–
Maculopapular rash	+++	++	+++	++	+++	+++
Itching	+	+	+	++	+++	++
Edema in hands	+	–	–	++	–	++
Conjunctivitis	–	++	-	+	+	–
Adenopathy	++	–	–	–	–	–
Fever	+	+ (38°C)	+	++ (39°C)	++ (39.5°C)	–
Retroorbital pain	++	–	–	–	–	–
Myalgia	NA	–	+	+	+	+
Laboratory						
Leukopenia	–	–	+	–	NA	+
Thrombocytopenia	–	–	+ (123 g/L)	–	NA	–
Activated lymphocytes	+	+	+	+	NA	+

*–, negative; +, light; ++, moderate; +++, intense; NA, not available.

To detect ZIKV in samples (RNA extracted from 200 μL of serum or urine), we used both sets of primers/probe specific for ZIKV (*11*). A standard curve with serial dilutions of known concentrations of a ZIKV virus stock was used to estimate viral load in samples. All blood samples were also tested for dengue virus and chikungunya virus by real-time RT-PCR and showed negative results. ZIKV was detected in serum of 4 patients (Figure). Urine samples from 2 other patients were also positive for ZIKV, and showed a higher viral load than corresponding serum samples and were positive for ≤7 days (patient 4) and probably >20 days (patient 3) after viremia reached an undetectable level (Figure). Urine samples from 6 healthy patients were also assessed and showed negative results.

**Figure:**
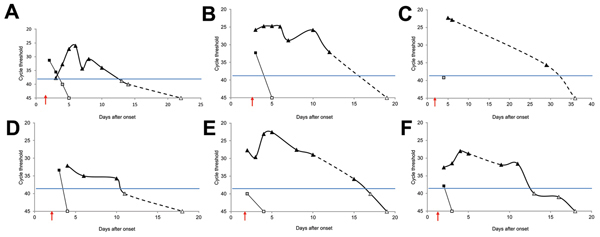
Detection of Zika virus in blood and urine specimens of 6 patients by using real-time reverse transcription PCR with primers/probe 1086/1162c/1107-Cy5 (*11*) New Caledonia, 2014. A) Patient 1; B) Patient 2; C) Patient 3; D) Patient 4; E) Patient 5; F) Patient 6. Triangles indicate urine samples and squares indicate serum samples. The cutoff cycle threshold (C_t_) value is 38.5, as previously reported (*11*) and is indicated by blue horizontal lines. Black symbols indicate amplifications with C_t_ <38.5, gray symbols indicate amplifications with C_t_ ≥38.5, and white symbols indicate negative amplifications. Onset of disease (day 0) was defined retrospectively after questioning patients about initial symptoms. Dashed lines indicate a period >2 days without a sample being obtained. Arrows indicate onset of rash.

Partial sequences of the gene for ZIKV nonstructural protein were obtained (*12*) directly from amplification products from urine or serum samples. Sequences obtained (GenBank accession nos. KJ873160 and KJ873161) had 100% identity with the sequence of a ZIKV strain isolated from a patient who returned from French Polynesia in 2013. As observed previously (*9**,**13*), sequences also had 98% identity with sequences of ZIKV strains isolated in Cambodia in 2010 and in Yap in 2007.

## Conclusions

We report the suitability of urine samples for diagnosis of ZIKV infection by showing that ZIKV RNA is detectable in urine at a higher load and with a longer duration than in serum. ZIKV infection has been poorly described because it is a benign, self-limiting illness in most cases (*5*). Thus, ZIKV infection has probably been underdiagnosed and underreported in disease-endemic settings (*4*) or in returning travelers. However, if perifocal vector control is to be implemented and severe neurologic complications are to be avoided, biological confirmation of ZIKV infection is essential. Because of the absence of specific IgM-based diagnostic tests, molecular confirmation is the only method available for routine diagnosis.

For ZIKV infection, date of onset of illness is difficult to establish because of sporadic and frequently mild fever. Although rash has been reported 3–5 days after the febrile phase (*6**,**7*), the 6 patients in our study had light asthenia and mild fever 2–3 days before the rash was observed; these symptoms were considered indicative of disease onset. Therefore, at the time the rash was observed, viremia was probably decreasing, which makes detection of virus in serum samples extremely challenging (Figure).

Other groups have reported that other flavivirus genomes, such as those of dengue virus (*14*), West Nile virus (*15*) and recently ZIKV (*13*), can be detected in urine samples for a longer time than in serum samples. Furthermore, use of urine samples for laboratory testing has some advantages, such as noninvasive sampling and ease of use. We detected ZIKV RNA in urine samples from all 6 patients. Urine samples showed strongly positive results; estimated maximum viral load was 0.7–220.10^6^ copies/mL. For all cases with sequential specimens, ZIKV RNA was detected ≤15 days (range 10 days to >20 days) after onset of symptoms, which was >7 days after it was not detected in serum samples.

In our study, ZIKV was detected in patient serum until a rash was observed (days 2–3 after disease onset). However, urine was preferred for virus detection. We observed a slight increase in ZIKV RNA from urine over the first few days after disease onset and rash (Figure). We therefore attempted to isolate ZIKV from urine samples, but failed to cultivate infectious particles. Further investigations are needed to evaluate whether live infectious ZIKV particles are excreted in urine, as has been observed for other arboviruses (*15*).

This study investigated the diagnostic utility of urine as a source for detection of ZIKV RNA by real-time RT-PCR. Results suggest that urine might be useful for confirmation of ZIKV infection because virus was detected at higher titers and for a longer period in urine samples than in serum samples. Although these results need confirmation in larger cohorts, they strongly suggest the suitability of urine as a specimen for ZIKV detection and screening in large-scale investigations or other epidemiologic contexts (e.g., returning travelers).

In industrialized regions, where local transmission of arboviruses, such as dengue virus or chikungunya virus has been reported, physicians should test patients who return from tropical regions for ZIKV when a case of dengue-like infection is suspected. Travelers might be a source of local transmission because *Ae. albopictus* mosquitoes are a competent vector for ZIKV (*3*).
